# News and Miscellany

**Published:** 1881-10

**Authors:** 


### NEWS AND MISCELLANY.

A New Veterinary Journal, called *La Presse Veterinaire*, has just been started in Paris.

The Siberian Plague (*Anthrax*) has been at work among the horses and cattle of Russia during the past season.

A Disease of the Eyes, causing total blindness, is reported to be affecting cattle in Lincoln, Ill. Its nature is unknown, or at least not reported.

A great number of horses at La Salle, Ill., are affected with a disease which, from newspaper reports, is presumably influenza. But few deaths are reported.

A Prize of $250 is offered by the Veterinary Medical Society of Paris for the best essay upon the following subject: Can pleuro-pneumonia be propagated by the use of the flesh of animals suffering from the disease?

Pimples, Whelps, Eruptions.—When this condition arises from an impure state of the blood benefit is always found from the administration of half a pound of Glauber's salt in a quart of water, and repeated in twelve hours if necessary.—*Southern Clinic.*

New Veterinary Medical Colleges.—It has been proposed to start a veterinary college in St. Louis, Mo. Measures are also said to be afoot looking to the establishment of a veterinary college in Philadelphia, under the direction of the University of Pennsylvania.

Pen and Plough.—We have to thank the *Pen and Plough* for a kindly and intelligent criticism upon our editorial on "Doctoring Through the Newspapers." The journal in question agrees with us in the main. There is, indeed, but one position that can be honestly taken upon the matter.

A Peculiar Colic appeared last July and August among the horses at Red Bank, Long Branch, Ocean Grove and other places in New Jersey. Fourteen fatal cases were reported, the animals generally succumbing to the disease in about one hour. In a few cases they dropped dead on the road.

Anthrax in Louisiana.—A form of anthrax has been affecting horses and mules in the vicinity of Pointe a-la-Hache, La. Swellings or tumors appear on different parts of the animals, which, if not early controlled, rapidly increase and cause death. The loss so far is about thirty-five head.

Alum for Cows in Summer.—It is a custom among our most prosperous German dairymen to drench their cows with a half pound of alum twice a week during the summer months. This is given to cool the blood and protect them from diseases incident to the summer season; at the same time it increases the flow of milk.—*Southern Clinic.*

An Item from Northern New York.—Our correspondent, Dr. G. Henry Kidney, of Malone, New York, writes: "We are having a great many cases of influenza (or pink-eye), also of tuberculosis in cattle. I have also had a case of tapeworm in the dog, caused by eating the bowels of the deer. The Journal of Comparative Medicine and Surgery is a perfect gem, and no veterinary surgeon should be without it."

The Old and the New English Race Horses.—The real difference between the old set and the new set of animals seems to be this: That whereas the earlier runners thought nothing of contesting three four-mile races in a week, and kept their power of doing this year after year, the modern flyer who accomplishes three miles once in his career and does not break down until after he has ceased to be a colt is considered a prodigy.

A Female Boa Constrictor ("Python Molurus") of East Indian extraction, it is hoped, is about to hatch out the first specimens of that snake that have ever been bred in captivity. The Regent's Park, London, Zoological Garden is the scene of the nesting. In 1862 an African python sat for ten weeks on her eggs, at the expiration of which time they were unfortunately removed, it appearing afterwards that a few more days would have seen the little serpents hatched.

Euphorbia Villosa is said to be regarded (Allgemeine Medicinische Central Zeitung, March 26, 1881) in the Ukraine and Gallicia, as an unfailing remedy against hydrophobia, when taken within five or six days of the infection. Unusually good evidence is claimed in its favor. It is strange that so many hydrophobia specifics start from Russia and prove valueless elsewhere. Erroneous diagnosis is most probably the cause for these therapeutic fiascos.—*Chicago Medical Review.*

Death of a Famous Jersey Cow.—Jersey Belle of Scituate (7,828), the property of Mr. Charles O. Ellms, of Scituate, Mass., died on August 11th, of milk fever, having dropped a bull calf the day previous. She was generally regarded as one of the most valuable Jersey cows in this or any country, and many connoisseurs pronounced her the best that has yet been produced. She was ten years old. The milk or puerperal fever is a trouble to which the closely bred Jersey cow is not unfrequently subject.

Exportation of Contagious Pleuro-pneumonia to England.—The report of Mr. Chas. P. Lyman, who was sent to England by the Commissioner Le Duc, to investigate the question of pleuro-pneumonia in American cattle, has been received. It shows that it is extremely doubtful whether single case of the real contagious pleuro-pneumonia has ever been exported from this country to Great Britain, and affords additional reasons why our government should ask to have the case re-opened by the British authorities.

A Curious Epidemic.—A singular disease, to which the veterinarians have not yet given a name, broke out among the cattle in Watertown, N. Y., last month. The stock attacked are blooded animals, and are in the very best condition. Several have died. They are taken first with intense coughing, followed by loss of appetite and shrinkage of milk. When let out where they can have perfect freedom they appear almost crazy, and will bite themselves, tearing out pieces of flesh, and appear in the greatest agony. The attention of the State authorities has been called to the matter.

Canadian Cattle Healthy.—Dr. Tache, Deputy Minister of Agriculture, referring to the reports that pleuro-pneumonia exists among the cattle of Nova Scotia, states positively that there is no cattle disease in the Dominion as is mentioned in the British order in Council regarding cattle importation.

New Veterinary Publications.—Dr. Hermann Pütz has published a work upon the Causes and Means of Extinguishing the Lung Plague. In it he gives the history of the abolition of this plague in Holland, and lays great stress upon the value of inoculation.

A valuable work on Veterinary Pathology, by Dr. Hill, is to be published soon by Mr. W. R. Jenkins, of this city. Also, Fleming's Veterinary Surgery, by the same publisher. The same gentleman is soon to publish Fleming's Veterinary Surgery. It will be a most valuable work.

A "Cure" for Hydrophobia.—Dr. Buison, of Paris, claims to have cured himself and several others of hydrophobia by a system of vapor baths. The directions are as follows: When a person has been bitten by a mad dog he should be made to take seven of the so-called Russian vapor baths, from 57 deg. to 63 deg. centigrade, hot, one every day, by way of preventive. In case of the malady having distinctly shown itself, the vapor bath should be heated rapidly to 37 deg. centigrade, then slowly to 63 deg. The patient should strictly confine himself to his room until he is quite well.

Gratitude.—At the recent presentation-day ceremonial at London University, Lord Granville, in his address, related the following anecdote, which we record as a contribution to the next work published on animal instincts Speaking of the Brown Institute, the noble earl said: "A distinguished member of the Senate was driving along the road in which the institution was situated, when suddenly his hack-cab came to a dead stop. He asked the driver whether his animal was lame or ill, but the driver answered, 'No, sir; I never can get him past this place since he had his corn cured here. He likes it so much that he always wants to stop.'"

Dr. Noah Cressy is the recipient of a compliment, never before extended, it is safe to say, to a veterinary surgeon in this country, in the shape of an invitation to deliver a course of lectures on Comparative Pathology before the Medical Department of Yale College. To extend the benefits of this course as widely as possible, the Faculty will invite the medical fraternity of the city, as well as the New Haven County Farmers' Club to be present. The first lecture of the course will probably be given early in October. Yale takes the initiative in this matter, being the first of our American colleges to recognize the importance of this branch in a medical course of instruction.

Mr. Graves, the Snake Charmer, on the Care of Snakes.—Mr. Graves believes that nearly all animals have reasoning powers, but thinks that snakes are devoid of such powers. He is sure that they cannot hear, as he has frequently tried to stir them up by making a great noise, and they would not budge. Their eyes are immovable. He was satisfied that a great many snakes die because their owners do not know how to handle them. He learned how to treat snakes from Mr. Barrett, of the Zoological Garden, Surrey. The handlers should frequently try snakes with food, as it was hard to tell when they wanted to eat, and they should have plenty of water.

MILK AS A CAUSE OF DEATH.—According to Dr. Brush (*N. Y. Medical Record*), the greatest cause of disease among infants in New York City actually arises from the use of diseased milk and the mixture of colostrum with sound milk. It may be considered positively dangerous to give a child milk when this milk has been drawn from a cow before the third or fourth day after calving. Nature then presents in the lacteal fluid of the cow a substance which is highly purgative, useful to her new-born calf, but certainly prone to give cholera infantum to a child. In cases of pleuro-pneumonia in cattle the milk is absolutely poisonous.

THE MANURE NUISANCE IN NEW YORK is the most troublesome problem that the Health Board has to solve. The secret of the trouble is that the manure is worth money, and that its price has been raised until its value is fictitious. The owners and keepers of horses are responsible for the nuisance, and they should be compelled to see that the manure that is produced leaves the city. At present the stable owners say that it is better to have one nuisance—the manure mound at the foot of East Forty-fifth street—than to prohibit further cartage of manure to it, and thus make a nuisance of every stable in the city. One thousand loads of manure are produced in New York every day. The Health Commissioners insist that it must not be allowed to accumulate either at the stables or at the dumps.

ENGLAND AND PLEURO-PNEUMONIA IN AMERICAN CATTLE.—The veterinary surgeon of the Department of Agriculture, who was sent to England in June last, by Dr. Loring, the United States Commissioner of Agriculture, to investigate the question of pleuro-pneumonia among American cattle landed in Great Britain, in connection with the Privy Council, has returned, and reports that upon his arrival in London, and at his solicitation, an important meeting of the Privy Council was held, the Lord President, the Earl of Spencer, presiding, and that the result of the examination and discussion of the subject which there took place has greatly tended to remove from the minds of the English authorities the strong impression they had formerly entertained as to the existence of contagious pleuro-pneumonia among cattle at the West.

A MONSTER TURTLE was brought on to this city not long ago It is of a variety never seen before in this market. It is seven feet long, four feet three inches broad, and about three feet thick. Its "flippers" or pectoral fins are forty-seven inches long. It is bluish black all over except on the neck, where muddy white spots, and under the throat pink spots, relieve the black surface. The back is marked by seven longitudinal ridges, there being one large ridge in the centre and three smaller ones on either side. The head is roundish and about a foot in diameter. The mouth is eight inches long and two long fangs protrude from the end of the upper jaw. These fangs are exceedingly sharp. Unlike most of the turtle family this specimen has not the power of drawing his head into the shell, and he is also incapable of walking, possibly because of his great weight, which is estimated to be between 1,700 and 2,000 pounds.

ANOTHER NEW CATTLE DISEASE.—The *Drovers' Journal*, Chicago, Illinois, says: "A strange disease has manifested itself among the cattle, in central portions of this State, that seems to be exciting more public mention than the facts would seem to warrant. The disease manifests itself in the animal's eyes, which become watery, then show signs of inflammation, and this followed by 'boil-like gatherings on the pupils of the eyes' We have heard of its outbreak in Menard, Logan, and McLean counties, and in two or three localities further south. A Menard county correspondent says, the most generally accepted theory as to the cause of the malady is that it is occasioned by the combined influences of heat, dust, and scarcity of water. Cattle grazing on timbered pastures do not appear to be subject to attack. Cattle-raisers recall the prevalence of the same disease in previous years of drouth and excessive hot weather. But slight damages resulted then, and it is believed that not much harm will result this year."

An Alligator Sunstruck.—One of the two alligators at Benton Park, St. Louis, was sunstruck recently—the first case of the kind on record, but as it was officially reported by the park commissioner, it will not be denied. The alligator turned over on its back and popped out its eyes and showed other unmistakable symptoms of sunstroke. The park keeper says it was a clear case. He had read of the way sunstruck persons are treated at the city dispensary, but he had neither ice nor ice water with him, and as it was a bad case, one that required immediate treatment, he pulled out his brandy bottle and gave Mr. Alligator a big dram. The effect was wonderful. The patient flopped over on his belly again and swam off, seemingly as happy as a big hungry catfish among a lot of little minnows. This is one of the pet alligators recently donated to the city by Democratic Gus, the saloon keeper on Seventh and Chouteau avenues, and it is said that he was raised on the bottle.

Phagedæna: Is it a New Disease?—In reference to this, Dr. A. G. Bolton, of Mankato, Miss., writes: "Phagedæna is not a new disease as Dr. Bowler seems to think, as you will find by consulting Robert McClune's work entitled *Diseases of the American Horse, Cattle and Sheep*, page 172, published by John E. Potter & Co., Nos. 614 and 617 Sansom street, Philadelphia, in 1870.

"The cause and symptoms given by McClune are the same as Dr. Bowler gave us in the July number of The Journal. I might copy McClune's account of the disease, but perhaps it would take more of your valuable space than you could give, although, if you do not succeed in finding a copy of the work mentioned, I will send it to you."

We believe it is thought in Chicago that the disease was equinia or horse-pox. *The Chicago Medical Review* discussed the matter quite extendedly at the time of its appearance.

Food for Wild Beasts.—At the Jardin des Plantes in Paris, the following are the allowances for the various animals. The monkeys are fed on carrots, cooked potatoes, salad, and bread made from maize and potatoes. The lions, tigers and bears have each five kilos. (including bones) of fresh meat per day. For the panther three to four kilos., and for the hyena, two to three kilos. of the same suffice. The wild cat gets half a kilo. of fresh meat without bones. The falcon gets the same. The eagle is served with one kilo., and the vulture with one-and-a-half kilo. of fresh meat with bones per day. The elephant eats per day four bundles of sainfoin, one decalitre of bran, two kilos. of bread, three or four bundles of oat straw; cost, 6f. The giraffe is supplied with one bundle of lucern, two pounds of bread, one litre of barley, one litre of beans, two litres of maize; cost, two and a-half to three francs. The stag, on a half bundle of lucern and five litres of bran, is kept for about one franc per day. Parrots and similar birds are fed for twenty-five to thirty centimes per day.

Splenic Fever in Dutchess County, New York.—A sudden and singular epidemic has lately broken out at Amenia, in this State. The first appearance of the disease was about the 9th of August, when a young and promising cow which had shown no previous symptoms of illness, was found in the morning dead in the pasture. Soon after this discovery another cow of the herd was seen to be slightly ailing, when the whole herd—about twenty in all—was put under the influence of carbolic acid exhalations. Coal tar was also freely used to cleanse and purify the stables, on the supposition that the disease might be one of contagion. In spite of all precaution, however, the sick cow died, another followed in her footsteps, and still another was found dead in the field. Post-mortem examinations showed the disease to be splenic fever or splenic apoplexy. Five cows in all subsequently died. The Governer and State Board of Health were informed of the existence of the disease.

The Horse and the Man.—The man has cut away the frog because he thinks that the animal will be injured if the frog touches the ground. He has then cut a deep groove at the base of the frog. This is to give a "well opened heel," as he is pleased to call it. He has scooped away the sole to "give it spring." He has scored a deep notch in the toe for the purpose of receiving the "clip" of the shoe. This is evidently a conservative relic of the time when nails were not used, and the shoe attached by three pointed clips hammered over the edge, one in front and one on either side. Then he has improved the whole of the outer surface of the hoof. As the Creator has furnished this part of the hoof with a thin, hard, polished plate, forming a sort of varnish which is impervious to wet, the farrier, as a matter of course, rasps it all away up to the crown. And as the Creator has placed round the crown a fringe of hair which acts as a thatch to the line of junction and throws off the rain upon the water-proof varnish, he cuts this away with his scissors. Lastly, the Creator having given to the horny hoof a mottling of soft, and partially translucent, brown, gray-blue, yellow, black and white, never exactly the same in two hoofs, much less in two horses, the farrier takes a blacking-pot and brush, polishes up the hoofs until they look like patent-leather boots, all four exactly alike, and then contemplates his work with satisfaction. In his own words, he has "turned out a finished job of it."—*Good Words.*

The Epizooty Again.—The annual appearance of the influenza or epizooty among horses in the early autumn has become as much a matter of course as the attacks of hay fever on some people. This year an old name has been revived for the disease, viz, "pink eye;" but the symptoms of the malady are not much different from those of former years. The animal's legs begin to swell, the eyes become red and discharge matter, then the tonsils become congested, and a cough appears. Loss of appetite and a generally dejected appearance also accompany the disease.

Veterinary surgeons vary in their ideas of the proper treatment, but the general policy is to give the animal rest, guard against cold, and allay any febrile symptoms; blisters are also used to reduce the swelled limbs and mustard plasters are applied to the neck and chest. Some doctors try to induce a discharge from the nose, which they claim relieves the inflammation in the eyes, by steaming the animal's head.

The disease has been noticed in Chicago and other Western cities for some weeks. Within a week or two it has manifested itself in this city. Probably few large stables are without one or more cases, though many of the stable-keepers do not notice it, as the disease has assumed a very mild form so far.

Veterinary Education.—The amount of education possessed by some of the practicing "veterinary surgeons" of Chicago must be rather limited, to judge by a recent occurrence (*Druggist*, September, 1881). One "veterinary surgeon" recently wanted four ounces of tincture of aconite root from a pharmacist, whose curiosity led him to ascertain the fact that the "veterinarian" believed that tincture of aconite root and "sweet spirits of nitre" were one and the same thing.

The Horse Disease in St. Louis.—There are 25,000 horses in the city of St. Louis, and at the present time, according to the best reports attainable, about one out of three of them is laid up with the disease which is called "pink-eye." The disease is rapidly spreading, and in its progress much resembles the epizooty which swept over the country from ocean to ocean in the winter of 1872 and 1873. It is not as fatal in its effects as the epizooty. The stablemen seem to have combined to keep the public in ignorance of the existence of the disease. It showed itself here about four weeks ago. It is known to be in existence at the present time in nearly all the large cities of the Union. Its exact nature is unknown, and its origin is as profound a mystery as that which surrounds the epizooty. The veterinary surgeons are studying it carefully, and are succeeding in reducing the number of fatal cases.

The above is a newspaper account of the trouble. It is probable that the disease is really only the epizooty.

A Veterinarian Selling Diseased Cattle.—A sensation was caused in Camden, N. J., not long ago by the report that William B. Miller, a veterinary surgeon and an employee of the State Board of Health, had been engaged in bringing to the city and selling cattle affected with pleuro-pneumonia. It was alleged that Miller and a butcher in his employ had seized diseased cattle, driven them past herds of sound cattle, and sold some of them alive and others after they had been butchered. Dr. Miller was called on. He had just returned from Trenton, whither he had been summoned by the State Board of Health. He said the herd in question had been condemned and quarantined by order of the board, and the cattle offered to a number of butchers at the appraised price, but they refused to purchase. He regarded the meat as good for food when the disease was in its incipient stages, and said the medical men on the Board of Health concurred in the opinion. He admitted that the cattle were brought to Camden, but said it was by order of the Board of Health, and they were willing to take the full responsibility for their action. A large mob was present at Washington, which would have interfered had there been an attempt to kill and bury the cattle there. He added: "I apprehend no danger of the spread of the disease by the removal of the cattle, as they were not permitted to come within gun-shot of any others, and I had the car in which they were transported to Camden thoroughly fumigated when it was emptied."

A Strange Disease.—A strange disease has, it is reported, broken out among cattle in Nova Scotia, and the mortality among the animals attacked appears to be very high. The disease is pronounced to be non-contagious, and confined to the vicinity of towns; but what it is has not, apprently, been determined.

A subsequent examination of this disease by Dr. Thayer, of the Treasury Cattle Commission, showed it to be less formidable than was at first supposed. His description is as follows: The animal is dull, the coat staring, loss of appetite, secretion of milk diminished, the temperature rises, the pulse is rapid, compressible, not wiry. In five or six days diarrhœa sets in, with black stools—in a few cases, extreme constipation—and in two or three weeks death. A post-mortem examination showed the following:

The thoracic viscera were healthy, with the exception of a slight pleuritic adhesion, the result of a former pleurisy. The brain was normal, and the pleura was quite pale.

On opening the abdomen, a large quantity of serum, estimated at more than five gallons, escaped; the same pale appearance of the serous-membrane was found as was seen in the pleura; the organs were removed separately. The spleen was firm, and weighed one pound eight ounces; the liver was of average size, and firm; the gall-bladder was enlarged and distended with bile. A portion of the latter was dark. (The butcher stated that he had seen the gall-bladder twice as large, and filled with something as black as tar and as thick as molasses.) The whole digestive tract was laid open and examined, but no trace of disease could be found. The kidneys and bladder were healthy.

Regarding contagion, Dr. Thayer says: The question of contagion may be considered as an open one; the fact, as stated to me, that cattle mingle together in pastures during autumn months, and are exposed during the winter in barns, without any outbreak of the disease from August to late in June, would seem to point to other causes than contagion.

The Horse Fever.—The following interesting case was sent by Dr. H. G. Farnsworth, of Clinton, Iowa, to the *Medical and Surgical Reporter:*

In this locality a new epizoötic is prevailing. It has gone through all the large stables, and is attacking isolated horses, and has also extended widely among the farmers of the county. It has produced considerable mortality. The doctor's horse, in a country practice, is his constant companion, and becomes almost as dear to him as one of his family; therefore, when my equine became sick I watched him with the same solicitude that I did any other patient.

On the morning of August 14th I found one of his hind feet swollen and quite tender; attributed it to a scratch received several days before, and let him remain in the stable. In the afternoon his eyes were dull and filled with a muco purulent discharge; this also appeared in his nostrils. The next morning the discharge was profuse; both hind legs were swollen; refused his food, and seemed to swallow water with an effort; it did not seem on account of swollen glands as much as from a difficulty of deglutition and stiffness of the jaws; gave him a small apple, which he dropped, because he could not get it between his grinders. Using my fever thermometer, I found his temperature 105°; led him into the lawn in front of the house where there was some tender grass; he seemed to put his head to the ground with difficulty, and after trying it a few times desisted, and walked to me for sympathy, lifting his hind feet with difficulty and looking dejected. His bowels were loose and his water free. Having a bottle of fl. ext. of verat. virid. in my pocket, it occurred to me to try it, which I did, for the fever. I poured two drachms into some water, which he drank in a little time, with frequent sips; it cooled the fever sensibly. The next morning the fever was as high as the day before; gave the verat. vir. in the same dose, several times during the day: he took no food, but quite a quantity of water; he had evidently stood up all night, and, in fact, did so for several days and nights afterwards; the discharge from the eyes and nose became copious.

The fever was less the third morning; thermometer marking 100°; the stiffness seemed passing away, and he tasted grass and took a few oats. He did not eat very freely before the fifth or sixth day, or lie down. The discharge then ceased, the swelling disappeared, and he walked with no difficulty. He had lost flesh very markedly, and for ten days after seemed to be weak and sweat profusely when exercised. It is evidently a contagious, self limited fever, its period of incubation, from five to eight days, and it runs its course in about five days, having a period of high fever for three days. In some instances there was much swelling of the parotids, but no cough; the jaws became very stiff, and deglutition so difficult that in some cases no fluid could be swallowed for three or four days. The fever sometimes seemed to produce delirium, as the horse would dash its head wildly about, and require restraint to prevent injury. In some cases death occurred from swelling about the throat, but in most cases it happened in the height of the fever, or when exercised too soon after it was over, or during convalescence. In all cases where the stereotype horse remedies were used, bleeding or purging, death or prolonged convalescence resulted. Young and old take it, but debility and disease, of course, added to its fatality. There is no exemption in any stable or collection of horses, but some that are isolated with care have escaped it. On farms it seems to be general. It has appeared in various parts of the State, and seems to be spreading widely. The mortality is very small, and the best treatment the expectant one, or a careful letting alone. In my case, I have no doubt the fever was modified by the ver. virid., which, if not curative, decreased the suffering of the animal.

A Curious Deformity is described by Dr. A. H. Baker in the *Veterinary Review*. The subject was a horse, buckskin in color, rising six years old, fifteen hands and three inches high. The peculiarity lies in the generative organs, having somewhat the appearance of an hermaphrodite, but the development of the parts is unusual even in such cases. The vulva of the female organ is natural in outline, but the lips are grown together, leaving an opening at the bottom large enough to allow the male organ to pass out, the lower part of the labia majora forming the sheath of the penis, although it encloses only a small portion of it, leaving the glands always exposed and protuding at the point occupied by the clitoris of the female. The glans is perfect in every respect, but small, having the urethral fossa, cul de sac and fungus-like protuberance of the male. The mammary glands are entirely wanting, but two small teats are found in their natural position. The animal has the general appearance and *finesse* of the mare, but has never shown any indications of being in heat; it has the organs as far as micturation is concerned, of the male, but no testicles are visible, and never shows any inclinations of the stallion when in the company of mares in heat, but minds his own business like a gelding. The urine is passed in a small, steady stream, as with the male, but he takes the position of the female. He is perfect in every other way, and can trot in 40 without training. The glans penis that is exposed is about the size of the same organ of a gelding.

Animal Pests in Greenwood Cemetery.—The New York *Sun*, citing the report of the trustees of Greenwood Cemetery for 1880, says: It is noted that chipmunks made their appearance in the cemetery during the last year for the first time. The injury done by them has been deemed a sufficient cause for their extermination, and 2853 of them were killed. Ground mice to the number of 375, 148 cats, 40 dogs, 133 snakes, 24 moles and 54 rats have also been killed.

A Nervous System has been discovered by Dr. Chun in the *Velellidæ*, a family of Siphonophores; it consists of a quite typically differentiated plexus of branching and intercommunicating ganglion-cells.

A Good Move.—It is understood that in accordance with the urgent request of the Treasury Cattle Commission, backed up by the strongly expressed opinion of the leading cattle dealers at the Chicago Stock Yards, Gov. Cullum, of Illinois, will issue a proclamation forbidding any cattle from being brought into this State from the regions known to be infected with contagious pleuro-pneumonia, in accordance with Section 4 of the act of the last session of the General Assembly of the State.

Treatment for Scab.—In the course of a long article on "Diseases of Domestic Animals," in a late number of the *North Pacific Rural Spirt*, we find the following remarks about treating sheep for scab: Sheep scab, after long continuance, is thought by some practical men to become constitutional. It is hardly necessary to point out that the disease is purely a local one, and only affects the system in the same way that any continual irritation would affect it. Naturally, when the parasites are very numerous and extend over a large surface, it is difficult to apply remedies in such a manner that all the mites, and also all their ova, shall be at once destroyed. The destruction of a large number affords so much relief that it is not difficult to believe that the disease is cured; meanwhile the ova which are left uninjured quickly produce another brood, and in a short time animals are as bad as ever; thus the flockmaster gets the impression that the affection has got into the system and cannot be got rid of by local remedies, the truth being that the treatment fails, simply because it is not properly followed up.

Evolution and Hybridism in Ornithology.—In a review in *Nature*, of the British Museum Catalogue of Birds, vol. fifth, containing the family *Turdidæ*, by Henry Seebohm, it is stated that one great feature of the work is the courage which the author has shown in applying the doctrine of the evolution of species to the birds as they exist at the present day. The great risk that the reviewer sees in Mr. Seebohm's method lies in the fact that it affords too easy a solution for otherwise difficult problems, but we must remember that the author was himself witness to the inter-breeding of the carrion crow and the hooded crow in Siberia, and it is known that this also takes place in certain parts of Great Britain. Having seen this with his own eyes, and brought back to England a large series of the hybrids, it was only reasonable for him to suppose that other birds are also capable of hybridizing, and the reviewer thinks that the author proves his case with regard to the two blue rock thrushes (*Monticola cyanus* and *M. solitarius*), which in certain parts of China interbreed; and it is most curious that the vast majority of the birds found in the winter quarters of the Eastern blue rock thrush, from Burmah and Malaisia to the Mollucca islands, appear to be hybrids. According to the author, *Cettia cantans* and *C. minuta* also interbreed and produce an intermediate form which he calls *Cettia cantans minuta*, a reintroduction of trinomial nomenclature which the reviewer does not at all like. The intermediate form, too, appears to be principally found in the Island of Formosa, though also met with at Chefoo, on the mainland opposite Japan, while one of the other forms is an inhabitant of Japan, with the exception of one Formosan skin in the author's collection, and the other is said to breed in South China and Hainan. Of these three forms, then, we should suppose that the Formosan was the oldest bird from which the other two had developed themselves, but that they had not as yet become entirely separated as distinct species. We must wait for more evidence with regard to the South African chats, to some of which Mr. Seebohm has applied his principle of hybridization, as the reviewer is not yet satisfied that the changes of plumage cannot be accounted for by the more natural process due to age or the season of the year.

"FIRING HORSES."—Why is firing horses' legs so persistently continued? Is it, like bleeding formerly was, from habit, from popularity, or does it possess the curative power ascribed to it? Two hundred years ago, firing was said to cause diseased bone and humors to die or crumble away; now it is said to produce a permanent bandage, a theory about as tenable as the former. The operation itself consists in burning the skin with a red-hot iron, often on a considerable surface of two legs at the same operation, and no one, whether professional or otherwise, can question for a moment that it is a prolonged, most painful, and cruel operation—nay, I may say barbarous; and should only be undertaken as a necessity, or with a reasonable certainty of success, and not as, is too often the case, as a last resource, or a kill-or-cure remedy. It is also often performed where no disease exists, but professedly as a preventive, by others for every form of disease or weakness. By some so lightly as to penetrate the outer surface of skin only; by others through the entire skin, which in so far as pain is produced, matters little, since the most sensitive part is between the outer and inner, or true skin.

Is firing successful? Methinks that if the mutilated horses found working the plough, the cab, and the costermonger's cart were placed beside those still retained for the carriage, the road, and the field, and if from the latter we withdrew those which had no organic disease, or only such of a simple or temporary character, it would not be flattering to the wisdom of the present day, and might well go where bleeding has gone, as a memorial of the prejudice of the age.

What is the action of firing? It is said to first produce much superficial inflammation; secondly, a permanent bandage. To produce a greater inflammation to overcome a lesser, is a doctrine happily dying out, and if necessary, firing is a severe way of applying it. The chief claim, however, to firing is the permanent bandage theory, or the production of a thickened, inelastic, malformed skin, exceedingly sensitive to the action of strain, cold, or injury, which depends in extent as to how much of the dermis or true skin is destroyed. If the outer or only the surface of the inner skin be cauterised, it may entirely recover, and there consequently would be no permanent bandage, and if through at intervals, or done with a thin iron, the cicatrix would be so small as to affect only in line of fire; but if the firing be broad and deep the whole structure of the skin acted on would be permanently altered in character, preventing all elasticity in line and diminishing it transversely, which, if covering a joint, produces a rigidity of its action or a more or less anchylosed condition of the parts, and what permanent effect it may have is probably more due to this than any other cause.

During an active practice of twenty years, for the last ten I have not fired a single horse, although here in Bristol and neighborhood, being hilly, horses of all classes are severely worked. I have found mild and almost painless treatment more speedy, more effective, and more permanent.

In my opinion firing is unscientific. It is a cruelly painful and prolonged operation. Its results are very uncertain, and do not justify the operation. It considerably blemishes, and consequently depreciates the money value of as well as the owner's respect for his horse; and finally, firing seldom removes, often does not diminish, and sometimes increases the disease under treatment.—G. W. GREGORY, M.R.C.V.S.

TAPEWORM.—The origin of these parasites in a large number of lambs is often a mystery. As the *cysticerci* of the tapeworm found in the lambs are not, like measles, found in the muscles of vertebrated animals, it is considered (by Cobbold) to be almost certain that some of the small molluscs and insects found in water, or in damp situations, constitute the intermediary bearers of the tapeworm larvæ in question. The tapeworm inhabiting the lambs is the *Tœnia expansa.* The lambs thus affected may have been inhabited by tapeworm some length of time before any symptoms of ailment are noticed; for it is a slowly-progressing disease. Amongst the remedial measures may be recommended the removal of the whole flock to a different pasture, where the ground is high and dry, and where provision should be made for ample and comfortable shelter. The weakly animals should be supported by oatmeal gruel or wheaten-flour gruel. Lambs suffering from scours should have a dose of one or two ounces of linseed oil, with two or three drops of croton oil, mixed, and given in a gill of warm gruel. Three hours subsequently, give one tablespoonful of the following cordial mixture: Prepared chalk, two ounces; catechu, one ounce; ginger, half an ounce; opium, one drachm: peppermint water, one pint. Various remedies are resorted to for the expulsion of tapeworms. Only a few are known to be reliable or effective. Among these are, kamala, kousso, koussin, oil of male fern, oil of turpentine, picate of potassium, etc. Two drachms of the kousso, in a gill of linseed tea or gruel, may be given every other day, for a few times. The koussin is still better, and may be given in two-grain doses, in a gill of wormwood decoction, twice or thrice, with two or three days between the doses. One dose of ten to twenty grains of the picrate of potassium may be given in half a pint of linseed tea, and should be f llowed, five hours thereafter, with a dose of physic, such as an ounce of castor oil, a scruple each of powdered ginger and salt of tartar, and an ounce of treacle. Oil of turpentine may be given in drachm doses, in a gill of linseed tea or gruel, once a day for a few days. Powdered squill has been recommended, in five to ten grain doses, with a teaspoonful of powdered ipecacuanha, given in gruel, twice or thrice daily. To expect success from any remedy, the animals should be subjected to treatment before the disease has so far advanced that a cachectic state or general emaciation exists. To ensure expulsion of the tapeworms after the administration of kousso, koussin or the picrate of potassium, the animals should not be fed the evening preceding the administration of the medicine early in the morning; and during the day of treatment, they should be given only a little hay, or, better, water into which is stirred some wheat flour. The tapeworms, as well as the fæces, passed by the lambs, after the medicine has been given, should be carefully gathered, and placed in some vessel, for the purpose of complete destruction by fire. The lambs should be liberally fed and comfortably housed. Change of situation, change of field, change of food are essential requisites in such a state of affairs as the one under consideration.—PAERIN, in *National Live Stock Journal.*

